# Anticipating pulmonary complications after thoracotomy: the FLAM Score

**DOI:** 10.1186/1749-8090-1-34

**Published:** 2006-10-06

**Authors:** Francesco Leo, Nicolas Venissac, Daniel Pop, Marylene Anziani, Maria E Leon, Jérôme Mouroux

**Affiliations:** 1 Thoracic Surgery Department, Nice University Hospital, Nice, France; 2 Physiotherapy Department, Nice University Hospital, Nice, France; 3 Division of Epidemiology and Biostatistics, European Institute of Oncology, Milan, Italy

## Abstract

**Objective:**

Pulmonary complications after thoracotomy are the result of progressive changes in the respiratory status of the patient. A multifactorial score (FLAM score) was developed to identify postoperatively patients at higher risk for pulmonary complications at least 24 hours before the clinical diagnosis.

**Methods:**

The FLAM score, created in 2002, is based on 7 parameters (dyspnea, chest X-ray, delivered oxygen, auscultation, cough, quality and quantity of bronchial secretions). To validate the FLAM score, we prospectively calculated scores during the first postoperative week in 300 consecutive patients submitted to posterolateral thoracotomy.

**Results:**

During the study, 60 patients (20%) developed pulmonary complications during the postoperative period. The FLAM score progressively increased in complicated patients until the fourth postoperative day (mean 13.5 ± 11.9). FLAM scores in patients with complications were significantly higher (p < 0.05) at least 24 hours before the clinical diagnosis of complication, compared to FLAM scores in uncomplicated patients. ROC curves analysis showed that the cut-off value of FLAM with the best sensitivity and specificity for pulmonary complications was 9 (area under the curve 0.97). Based on the highest FLAM scores recorded, 4 risk classes were identified with increasing incidence of pulmonary complications and mortality.

**Conclusion:**

Changes in FLAM score were evident at least 24 hours before the clinical diagnosis of pulmonary complications.

FLAM score can be used to categorize patients according to risk of respiratory morbidity and mortality and could be a useful tool in the postoperative management of patients undergoing thoracotomy.

## Background

Pulmonary complications represent the main postoperative problem following lung resection and have been the primary cause of postoperative morbidity and mortality over the last thirty years [1-3]. The use of standardized guidelines for the preoperative functional assessment [4] identifies patients at higher risk for complications but these patients represent only a select subset of the population who experience postoperative morbidity and mortality [5,6]. In other words, in the overall population of patients who undergo lung resection, the vast majority of pulmonary complications occurs in those patients preoperatively defined as not at risk.

The only tool to anticipate the occurrence of respiratory complications in these patients remains the surgeon's experience. Given the fact that pulmonary complications are delayed, usually occurring 48–72 hours after thoracotomy [7], and result from progressive changes in respiratory status, the standardization of postoperative respiratory assessment might permit earlier detection of respiratory changes that likely anticipate the clinical diagnosis of complications. To verify such hypothesis, a scoring system (the FLAM score) was developed in 2002 and prospectively applied to compare the respiratory pattern after thoracotomy in patients developing respiratory complications and in uncomplicated patients.

To assess the utility of the FLAM score in predicting respiratory complications, we conducted a study was to analyse respiratory status of patients who underwent thoracotomy by the daily use of the FLAM score.

## Methods

The study was a prospective study designed to enroll 300 patients undergoing posterolateral thoracotomy at the Thoracic Surgery Department of the Nice University Hospital, France. It was approved by the Internal Review Boards. Patients were considered eligible if 1) they underwent posterolateral thoracotomy; 2) they had epidural analgesia; and 3) pain control was effective (defined as a visual analogic scale -VAS-assessment ≤ 35 at rest and ≤ 60 during physiotherapy) 4) the informed consent was obtained. Patients having two VAS measurement at rest ≥ 35 or one VAS measurement ≥ 60 during physiotherapy in the considered day were excluded from the protocol.

During the study period, the score was only observational. No diagnostic or therapeutic decision was taken on the basis of the FLAM score. No study-specific procedures were performed and all the assessments performed were part of routine clinical care.

The surgical procedure was performed through a posterolateral thoracotomy with section of the latissimus dorsi muscle. After surgery, all patients were admitted to the high-dependency unit for 24–48 hours. Epidural analgesia was maintained until the 5th postoperative day (POD) and then replaced with subcutaneous morphine injection. After short term antibiotic prophilaxys, no routine antibiotic treatment was started after thoracotomy. The first chest drain was usually removed on POD 3, and the second on POD 5 if possible (fluid < 200 cc/24 hours, no air leak).

Patients had two 15-minute sessions of chest physiotherapy daily for the first 7 postoperative days. Level of pain was assessed by the staff nurse on the basis of a visual analogic scale 5 times/day. All patients had daily FLAM score assessment for the first 7 postoperative days. The first partial evaluation of the FLAM score was performed during the ward rounds by the surgeon and was subject to modification during the day when further evaluations were obtained.

### The FLAM score

The FLAM score was developed at the Thoracic Surgery Department of the University of Nice, France, by two of the authors (FL, MA) whose initials gave the name to the score. The parameters of the FLAM score were chosen based on a retrospective analysis of the Thoracic Surgery Department database performed in March 2002 and on analysis of data from a small pilot trial initiated in May 2002. The final FLAM score parameters were defined in September 2002, and all participating staff members were trained in their use before study initiation.

The FLAM score is composed of 3 main parameters (dyspnea, chest X-ray, and administered oxygen) and 4 minor parameters (quantity of bronchial secretions, quality of bronchial secretions, cough, and pulmonary auscultation).

Dyspnea was defined as a respiratory rate ≥ 20/minute lasting more than 2 minutes or associated with a decrease in pulse oximetry ≥ 10% from the last value recorded. Three different scores were possible for this parameter. The dyspnea score was 0 when no dyspnea was present; 5 when dyspnea was present only during chest physiotherapy or active mobilization; and 10 when dyspnea was present at rest.

Chest X-ray was scored as follows: the score was 0 when no abnormality was present, and 5 if lobar atelectasis or pneumonia was present. Complete atelectasis, partial atelectasis after pneumonectomy, pneumonia involving the entire lung, and bilateral pneumonia were scored as 10. In cases of pneumonia, radiological anomalies were considered only when clinical criteria of pneumonia were met (see section "Pulmonary complications").

The oxygen rating corresponded to the highest rate of oxygen delivery (in number of liters of oxygen/minute), to a maximum of 15 liters/minute, delivered over the previous 24-hour period to maintain a hemoglobin saturation ≥ 94% (range 0–15) as recorded by pulse oxymetry. Oxygen scores ranged from 0 to 15.

Minor parameters evaluated by the FLAM score were rated on a scale of 0–2. The quantity of bronchial secretions was rated 0 if less than 5 ml/24 h, 1 if between 5 and 10 ml/24 h, and 2 if greater than 10 ml/24 h. The score for bronchial secretion quality was 0 for absent or mucous secretions, 1 for mucopurulent secretions, and 2 for purulent secretions. Efficient cough was scored 0, partially ineffective cough was scored 1, and inefficient cough was scored 2. Chest auscultation was rated 0 when no anomaly was present, 1 when secretion soundings were resolved after cough, 2 when secretion soundings were always present.

The FLAM score for any given day was the sum of all 7 parameters. The maximal possible score for patients without intubation was 43. Intubated patients were scored at 45 by definition.

### Pulmonary complications

The following pulmonary complications were considered: (1) ARDS, defined as respiratory failure with acute onset, PaO_2_/fraction of inspired O_2 _< 200 mm Hg and bilateral infiltrates seen on chest X-ray, and pulmonary wedge pressure < 20 [8]; (2) ALI, defined by the same criteria as ARDS but with PaO_2_/fraction of inspired oxygen < 300 mm Hg; (3) pneumonia, defined by the presence of at least 3 of the following criteria: persistent lung infiltrate on chest X-ray, fever >38°, white cell blood count >10000/mm^3 ^or <3000/mm^3^, purulent secretions, documented presence of microorganisms on sputum or bronchoaspirate; (4) atelectasis, defined as lobar or whole-lung atelectasis requiring bronchoscopy, (5) pulmonary embolism documented by lung ventilation/perfusion scintigraphy or angioscan, (6) pulmonary edema; and (7) asthma, defined as an episode of bronchospasm associated to dyspnea and cough due to a transient global narrowing of the airways [9]. Respiratory failure was defined as the need for non-invasive ventilation, postoperative mechanical dependence > 12 hours, or reintubation.

### Statistical analysis

The FLAM scores in patients not developing pulmonary complications were compared to the FLAM scores in patients developing pulmonary complications by graphical representation. The FLAM scores were then separately analysed for each type of pulmonary complication occurring at least twice in the study. To search for early changes in FLAM scores in complicated patients, their scores at 24 and 48 hours before the pulmonary complication were compared to the scores from the corresponding days in uncomplicated patients (controls). Comparisons were made using Student's *t *test.

To determine whether the maximum FLAM score recorded for each patient was an independent prognostic factor for respiratory complication, respiratory failure or death, we used multiple logistic regression models including preoperative FEV1%, which is a universally recognized predictor of respiratory complications, and operation type, as the risk of life-threatening complications is higher after pneumonectomy.

Receiver-operating characteristics curves (ROC) were used to evaluate the performance of the FLAM score in identifying pulmonary complication following lung resection. Each unique FLAM value was used as a cut-point to calculate sensitivity and specificity estimates defining the curve and the area under the curve (AUC).

All analyses were done using SAS (SAS Institute, Cary, North Carolina). P values were indicative of a statistical significant association if the corresponding two-sided p-value was < 0.05.

## Results

Between November 2002 and October 2004, 321 patients were prospectively included in the study. Thirteen patients were excluded from the analysis because of insufficient pain control by epidural analgesia. Incomplete data were obtained for 6 patients, and 2 patients were excluded because they had intrathoracic hyperthermic chemotherapy after lung resection. Thus, the evaluable study population of the study consisted of 300 patients.

Clinical characteristics of the evaluable population are presented in table 1. One hundred thirteen patients developed one or more postoperative complications of any type (37.6%), and 11 patients in the study died (3.6%) in the postoperative period. Mean postoperative stay was 9 days.

**Table 1:** Clinical characteristics of the study population

	n	%	Standard deviation
**Sex**			
Male	221	73.6	
Female	79	26.4	
**Age (mean)**	61.9 (range 16–84)		12.4
**ASA score**			
ASA 1	62	20.6	
ASA 2	138	46	
ASA 3	90	33.4	
**Preop. FEV1%**	85.7 (range 36–136)		19.6
**Pathology**			
Lung cancer	216	72	
Lung metastases	23	7.7	
Carcinoid	5	1.6	
Benign	39	13	
Others	17	5.7	
**Operation**			
Pneumonectomy	33	11	
Lobectomy	201	67	
Bilobectomy	12	4	
Segmentectomy	9	3	
Others	45	15	
**Operating time (mean)**	111 (range 45–300)		35.6

Sixty patients had postoperative pulmonary complications (20%), and 19 patients developed respiratory failure. Nine patients died as a result of postoperative pulmonary complications. Pulmonary complications recorded were atelectasis in 31 patients, pneumonia in 17 patients, progressive respiratory failure in 5 patients, ARDS in 4 patients, pulmonary edema in 2 patients and pulmonary embolism in 1 patient.

**Figure 1:**
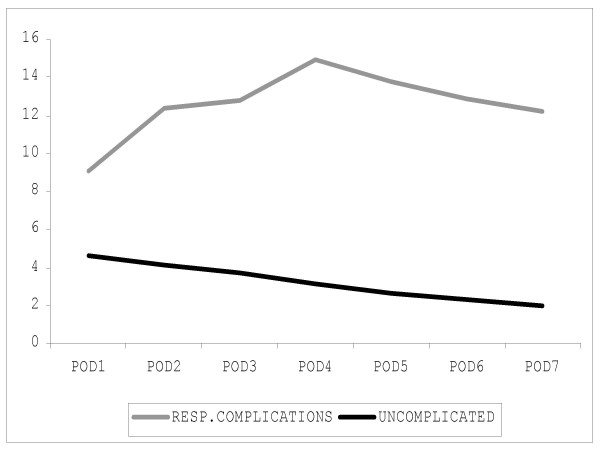
Patients developing respiratory complications (n = 60) showed a progressive increase of the mean FLAM score compared to uncomplicated patients (n = 240), who had a progressively descending FLAM. The difference was significant for all the 7 postoperative days.

**Figure 2:**
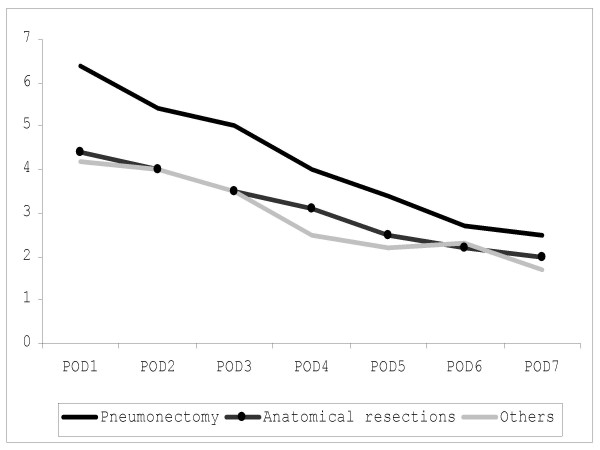
In uncomplicated patients, the mean FLAM score is moderately higher after pneumonectomy than after lobectomy, confirming the clinical impression that pneumonectomy patients are more delicate than lobectomy patients during the first 2–3 postoperative days.

Graphical depiction of the postoperative FLAM score in patients without pulmonary complication shows a descending curve with the highest value on POD1 and the lowest on POD7 (Figure 1). The FLAM score on POD1 was significantly higher (p < 0.05) in patients who underwent pneumonectomy than in patients who underwent lobectomy (figure 2). Differences between these two patient groups showed a borderline significance on POD2 and POD3 (p = 0.06).

**Figure 3:**
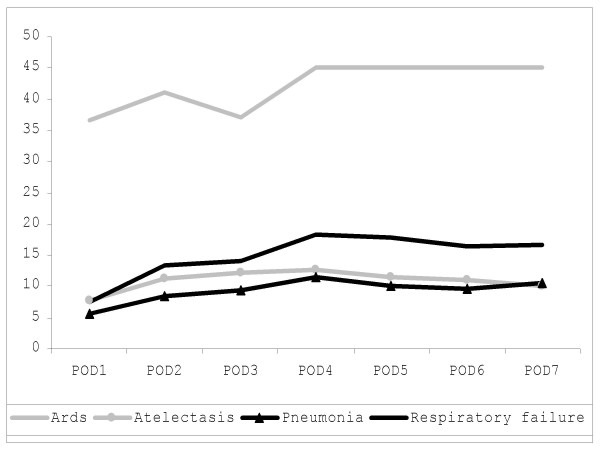
In complicated patients, the mean FLAM score increased during the first 4 postoperative days unregardless of the type of complication. ARDS patients raised rapidly to FLAM 45, which is the score of intubated patients by definition.

In patients who developed pulmonary complications, FLAM scores showed a progressive increase from POD1 to POD4 (figure 3). Compared to uncomplicated patients, patients who developed pulmonary complications had signficantly highly (p < 0.05) daily FLAM scores for the entire first postoperative week.

Analysis of pulmonary complications by the day of clinical diagnosis indicated that higher FLAM scores were evident at least 24 hours before the complication in all subgroups with pulmonary complications, compared to uncomplicated patients (table 2). In patients who developed complication on POD2 and POD3, this difference was evident event 48 hours before the event.

**Table 2:** FLAM variations in complicated and uncomplicated patients

	**Day of respiratory complication**				
	
**FLAM**	**controls**	**POD1**	**POD2**	**POD3**	**POD4**
	n = 240	n = 12	n = 15	n = 14	n = 10

**POD1**	4	14	7*	6.5*	5
**POD2**	4	15.5	13	8.5*	6.5*
**POD3**	3	16	10	10.5	7.5*
**POD4**	3	17	8.5	14	12
**POD5**	2	18	6.5	12	8.5
**POD6**	2	16	7.5	8	6
**POD7**	2	11	6	8	6

Dividing complicated patients by the day of clinical diagnosis of complication and comparing them to uncomplicated patients, an higher FLAM score was evident at least 24 hours before the event in all the subgroups (* p < 0.05).

**Figure 4:**
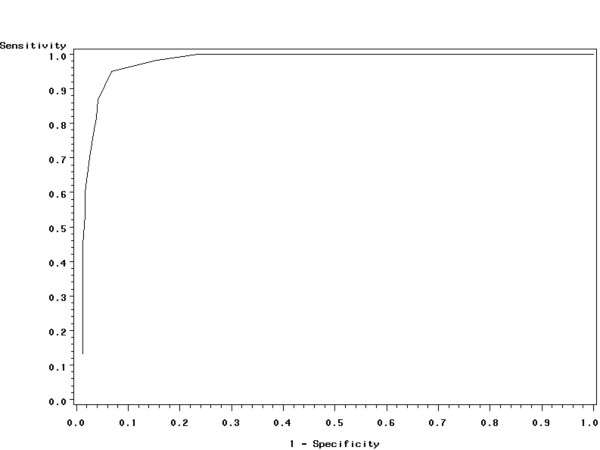
ROC curves indicated that the FLAM score value providing the largest sum of sensitivity plus specificity was 9 (area under the curve, AUC, 0.97).

ROC curves indicated that the FLAM score value providing the largest sum of sensitivity plus specificity was 9 (sensitivity 86.6%, specificity 95%, positive predicted value 81.2%, negative predicted value 96.6%, area under the curve 0.97, figure 4).

Patients were then divided into 4 groups based on the highest FLAM value recorded for each patient during the considered week using cut-off values of 9 and multiples of 9. The incidence of complications and mortality resulted progressively increasing from class I (maximal FLAM value 0–9) to class IV (maximal FLAM > 27, table 3).

**Table 3:** The 4 classes of risk defined by the FLAM score

**FLAM peak value**	**n**	**Pulmonary complications**	**Respiratory Failure**	**Death**
0 – 9	236	8 (3.3%)	0	0
10 – 18	42	32 (76.2%)	6 (18.7%)	1 (3.1%)
19 – 27	8	6 (87.5%)	4 (57.1%)	2 (28.7%)
> 27	14	14 (92.8%)	9 (69.2%)	6 (46.1%)

Considering the highest FLAM value recorded, it was possible to define 4 class with increasing risk of pulmonary complication and severity. It was called "the rule of 9" because class are identified by multiple of nine.

Multi-variable logistic regression showed that the class of FLAM had an evident impact on the risk of pulmonary complications, respiratory failure and death (table 4). Treating FLAM score as a continuous variable in the regression model and after controlling for preoperative FEV1% values, the analysis showed a 43% increase (confidence interval 28%–59%) in risk of respiratory complications after surgery for each unit increase in maximum FLAM score.

**Table 4:** Results from multivariate analysis

		ODDS RATIO (95% CI)		
		
**Factor**	**n**	**Respiratory complications**	**Respiratory Failure**	**Death**
**TYPE OF OPERATION**				
Pneumonectomy	33	1.39 (0.61–3.2)	4.26 (1.46–12.5)	5.99 (1.52–23.6)
Anatomical resection	222	1	1	1
Other	45	0.46	0.89 (0.19–4.2)	0.99 (0.11–8.6)
**Preoperative FEV1%**				
≥ 80%	**164**	1	1	1
< 80% – ≥ 50%	**72**	2.15 (1.13–4.1)	4.65 (1.42–15.3)	0.68 (0.13–3.6)
< 50%	**64**	6.45 (1.5–27.7)	13.3 (2–87.6)	4.54 (0.47–44.3)
**FLAM peak value**				
0 – 9	**236**	1	1	1
10 – 18	**42**	82.6 (18.8–364)	6.9 (0.6–7.8)	0
19 – 27	**8**	471 (72.7–999)	118 (13.4–>999)	38 (3.7–387)
> 27	**14**	999 (157–>999)	228 (25.7–>999)	111 (12–>999)

Multivariate analysis showed that the link between the FLAM highest recorded value and the occurrence of pulmonary complications is maintained even after adjusting for preoperative FEV1% and type of operation.

## Discussion

Even when accurate functional assessments are routinely performed before surgery, pulmonary complications remain one of the most important problems after thoracotomy. Interestingly, the complications are not immediate but are typically delayed until 48–72 hours post-surgery. During that time there is usually a progressive impairment of the respiratory function leading to the pulmonary complication [10]. If we could identify those patients at greatest risk for developing pulmonary complications 24–48 hours before their occurence, we could perhaps define more aggressive treatment protocols to reduce mortality. In this study, we assessed a tool designed to standardize the description of these respiratory changes in order to predict respiratory complication at least 24 hours before the event.

Study results were encouraging because the FLAM score clearly showed progressive impairment of patients before the complication. More interestingly, a significant increase in FLAM score was evident at least 24 hours before the event in all the complicated patients, regardless of the day in which the complication was diagnosed.

From the practical point of view, a FLAM value less than 7 is normal on POD1 after lobectomy. In case of pneumonectomy, the higher O2 needs of these patients can justify a slightly higher value (7-9). From POD2, progressive reduction in FLAM score is reassuring. The risk of complication increases if the score rises or fails to decrease after POD2. In this study, a FLAM value of 9 predicted the development of pulmonary complications with a sensitivity of 86% and a specificity of 95%. At the diagnosis of the pulmonary complication, the FLAM value is usually between 12 and 21, except for patients developing ARDS in which is usually higher.

Once the FLAM score has been recorded for a patient on a given day, the corresponding FLAM risk class (figure 3) indicates the risk of developing a pulmonary complication, assuming that the recorded value expresses the peak score for that patient. Patients with a FLAM score between 10 and 18 developed respiratory complications in 76% of cases, usually without impact on the length of the postoperative stay if the score descended the following day. Patients with a FLAM value higher than 19 developed a respiratory failure in 29% of cases. Postoperative mortality in patients with a FLAM higher than 27 was 46% in our series.

The early postoperative identification of patients likely to develop pulmonary complications may improve practice, as aggressive perioperative management can reduce postoperative morbidity and mortality, and expand the population that can receive potentially curative treatment [11]. Patients showing a progressively increasing FLAM score may have dedicated protocols of care such as an intensified physiotherapy program, coltural assessment of bronchial secretions, empiric antibiotics administration when signs of infection are present, high-dependency unit readmission when the score remains stably over the value of 10–15. The real advantage of this approach needs further studies to be confirmed.

In addition to this potential benefit, the use of this simple scoring system offers at least 2 other advantages. The first is an improvement in patient evaluation by inexperienced staff such as junior residents or low-volume thoracic unit staff. The second is the adoption of a common language between different centers, which in turn facilitates comparison of study results between different centers. For example, Auriant and colleagues reported a study evaluating patients on non-invasive ventilation in acute respiratory failure after lung resection [12]. Applying FLAM definitions to their inclusion criteria, only patients with FLAM > 19 were included. The overall mortality in the study was 25%, similar to the 30% mortality from the same category of patients in our study.

One of the most important limitations in the use of scoring systems is the intra – and inter-observer variation in score attribution. In the case of the FLAM score, this problem was limited by the definitions adopted. The agreement in score attribution between two different staff surgeons was tested by means of the κ statistic both in the first pilot study and in the first 100 cases from this series with an level of discrepancy < 10% (pilot study κ = 0.43 95% CI 0.40–0.52; present series κ = 0.50 95% CI 0.42–0.64).

Another limitation of the study is the decision to include only patients having good pain control by epidural analgesia. The importance of the variable "pain" on the occurrence of pulmonary complications [13-15] and the postoperative key-role of epidural analgesia [16] are well known. Our intent was to analyse the natural history of respiratory complications avoiding the masking effect of pain. The result is a very homogeneous population in which FLAM curve modifications were strictly related to the patient's respiratory status. In the planned prospective multicentric study on the score, FLAM evaluation will be performed on a cohort of 1000 patients in which the type of analgesia and effectiveness of pain control will not be considered as inclusion criteria.

In conclusion, the FLAM assessment is simple, intuitive and does not require additional procedures as compared to standard patients care in a thoracic surgery ward. It can represent an additional tool to improve our clinical knowledge in the field of postoperative respiratory complications in order to reduce their mortality. The FLAM score can be one more step towards achieving this goal.
